# Epidemiological and mechanistic links between PM_2.5_ exposure and type 2 diabetes: focus on the TRPV1 receptor

**DOI:** 10.3389/fendo.2025.1653375

**Published:** 2025-09-02

**Authors:** Filippo Liviero, Sofia Pavanello

**Affiliations:** Department of Cardiac, Thoracic, Vascular Sciences and Public Health, Occupational Medicine Unit, University of Padua, Padua, Italy

**Keywords:** particulate matter (PM), environmental risk factors, oxidative stress, neurogenic inflammation, autonomic nervous system dysregulation

## Abstract

The growing global burden of type 2 diabetes (T2D) has prompted increasing attention to environmental factors that may contribute to its development. Among these, exposure to fine particulate matter (PM_2.5_) has emerged as a significant yet often overlooked risk factor. This systematic review conducted according to the PRISMA guidelines, provides a comprehensive and critical appraisal of the epidemiological evidence and discusses mechanisms linking PM_2.5_ exposure to the onset and progression of T2D. Long-term exposure to PM_2.5_ has been consistently associated with increased T2D risk in epidemiological studies, particularly among vulnerable groups such as individuals with obesity, metabolic syndrome, or advanced age. In addition, evidence from animal models suggests that acute exposure can exacerbate insulin resistance and impair glucose metabolism. Mechanistic studies highlight the roles of oxidative stress, systemic inflammation, endothelial dysfunction, and autonomic imbalance. Notably, recent findings implicate the transient receptor potential vanilloid 1 (TRPV1) in neurogenic inflammation and metabolic disruption, offering novel insights into how PM_2.5_ may influence glycemic control. Experimental evidence in humans indicates that traffic-related PM_2.5_, including diesel exhaust particles (DEPs), activates TRPV1, supporting its role as a molecular interface between environmental insults and metabolic disruption. Given its central role in neurogenic inflammation and metabolic regulation, TRPV1 has emerged as a promising therapeutic target. Preclinical studies have shown that pharmacological modulation of TRPV1 improves glucose tolerance and reduces inflammation. Currently, XEN-D0501, a TRPV1 antagonist, is undergoing clinical trials to assess its efficacy in regulating blood glucose and mitigating T2D-related inflammatory complications. These mechanistic insights are further supported by animal studies demonstrating that PM_2.5_ exposure induces metabolic dysfunction consistent with TRPV1 activation and inflammation-related pathways. Animal models corroborate human data, revealing that PM_2.5_ exposure promotes visceral adiposity, impairs hepatic insulin signaling, and triggers tissue-specific inflammation. Despite the strength of the overall evidence, heterogeneity in exposure assessment, driven by spatial and temporal variations in PM_2.5_ sources and composition, and in study design persists. Given the ubiquity of PM_2.5_ in urban environments, even modest increases in diabetes risk may translate into substantial public health burdens. Targeted policies to reduce air pollution, together with intensified research into biological susceptibility and prevention strategies, are essential. Addressing PM_2.5_ as a modifiable determinant of T2D represents a timely and actionable priority in environmental health.

## Introduction

Air pollution represents a silent yet devastating global crisis, with consequences extending far beyond respiratory diseases, profoundly affecting public health and the environment. Among various pollutants, fine particulate matter (PM_2.5_) stands out as one of the most dangerous for human health. This microscopic pollutant, which is primarily generated by fossil fuel combustion, industrial processes, and vehicular traffic, penetrates deep into the lungs and circulatory system, triggering systemic inflammation, oxidative stress, and metabolic alterations. The health implications are alarming. PM_2.5_ is recognized as an independent risk factor for the onset and mortality of T2D. According to the Global Burden of Disease Study 2019 (1), 20% of global T2D cases are attributable to long-term exposure to PM_2.5_, with the impact being particularly severe in low- and middle-income countries. In Europe, the Burden of Disease 2024 by the European Environment Agency (2) identified T2D as the leading chronic degenerative disease linked to fine particulate pollution, followed by lung cancer, chronic obstructive pulmonary disease (COPD), and asthma. In Italy, the situation is particularly concerning. In 2019, the United Nations Environment Programme (UNEP) report (3) indicated that the annual average concentration of PM_2.5_ reached 16 µg/m³, which is more than three times the limit recommended by the World Health Organization (WHO). This exposure accounted for 24,666 deaths, with 14% of T2D-related fatalities directly attributable to PM_2.5_. This review aims to critically evaluate the scientific evidence linking atmospheric particulate exposure to the onset and progression of T2D, delving into the underlying pathogenic mechanisms and identifying the most vulnerable populations. Emerging evidence suggests that PM_2.5_ exposure induces chronic low-grade inflammation not only in the lung but also systemically, with neurogenic mechanisms playing a pivotal role (4). Inhaled PM_2.5_ activates pulmonary sensory neurons that express the TRPV1 receptor, leading to the release of proinflammatory cytokines (5). These circulating mediators contribute to endothelial dysfunction and the systemic propagation of inflammatory signals to multiple target organs, including adipose tissue, skeletal muscle, liver, and pancreas. In the pancreas, local neurogenic inflammation, mediated by TRPV1-expressing sensory neurons, may impair β-cell function, reduce insulin secretion, and promote insulin resistance (5). These pathological effects are further exacerbated by oxidative stress, endothelial dysfunction, and autonomic nervous system imbalance, particularly via sympathetic overactivity, which collectively impair glucose homeostasis. Furthermore, PM_2.5_ particles can reach the brain directly via the olfactory nerve, bypassing the blood–brain barrier and induce oxidative stress and neuroinflammation in key regions of the central nervous system (6). These alterations may disrupt endocrine signaling and autonomic regulation, further contributing to metabolic and systemic dysfunction (4). Addressing the impacts of PM_2.5_ is not only a vital step in reducing the burden of chronic diseases but also an essential commitment to safeguarding the health of future generations.

## Selection criteria and literature screening process to identify studies on PM_2.5_ exposure and T2D

**Figure 1:**
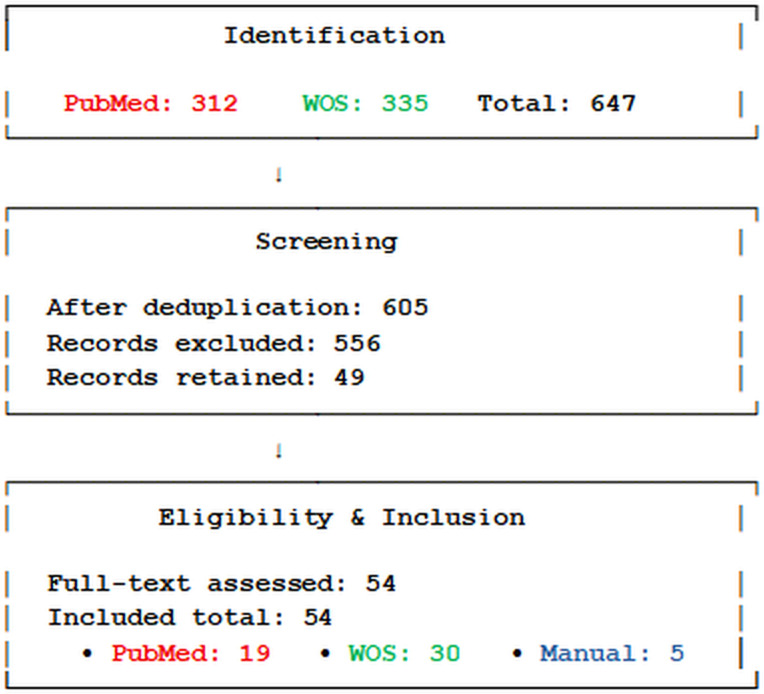
PRISMA flow diagram.

This systematic review was conducted according to the PRISMA (Preferred Reporting Items for Systematic Reviews and Meta-Analyses) guidelines (7), as summarized in **Figure 1**. A systematic search of epidemiological and animal studies, as well as systematic reviews and meta-analyses, was carried out using two electronic bibliographic databases: PubMed and the Web of Science Core Collection. The Boolean query (“PM_2.5_” AND “type 2 diabetes”) was applied to identify relevant literature published between January 1, 2005 and December 31, 2024. To ensure comprehensiveness, the PubMed search was extended to include publications from 2000 onward to capture seminal early studies. Records were limited to articles published in English. The initial search yielded 312 records from PubMed and 335 records from Web of Science. After removing 42 duplicate records found in both databases, a total of 605 unique citations remained for title and abstract screening. Titles and abstracts were independently screened by two reviewers based on predefined inclusion criteria: original epidemiological studies (cohort or cross-sectional) examining the association between long-term PM_2.5_ exposure and T2D or related glycemic outcomes; experimental animal studies investigating metabolic perturbations induced by PM_2.5_; and systematic reviews or meta-analyses on the same topic. Studies were excluded if they focused on pollutants other than PM_2.5_, lacked primary data (e.g., editorials, commentaries, conference abstracts), or did not report relevant metabolic endpoints. Any discrepancies between reviewers were resolved by discussion and consensus. This screening process excluded 556 records, leaving 49 studies from the databases. An additional 5 studies were identified manually through citation tracking, resulting in a total of 54 articles included for full-text review and qualitative synthesis. Of the 54 studies included, 19 originated from the PubMed search, 30 from the Web of Science, and 5 were manually added. All were peer-reviewed and met the inclusion criteria.

## PM_2.5_ exposure and onset of T2D

The relationships between T2D and PM_2.5_ exposure have been addressed in two aspects: the search for a “causal” association between PM_2.5_ exposure (short- and long-term) and T2D and the verification of the hypothesis that diabetic individuals are particularly susceptible to the short-term cardiovascular effects of PM_2.5_ exposure (8, 9).

## Associations between PM_2.5_ exposure and T2D

### Long-term exposure

#### Studies reporting positive associations between PM_2.5_ exposure and T2D

Long-term exposure refers to average exposure levels spanning from several months up to several years. **Table 1** consolidates findings from 28 epidemiological studies on the relationship between PM_2.5_ exposure and T2D. These studies, which span diverse geographical regions and employ various designs and populations, consistently report positive associations between both long-term and short-term PM_2.5_ exposure and increased risk of T2D.

**Table 1:** Principal studies supporting the positive association between PM_2.5_ exposure and T2D.

Study/reference	Study type	Population type	Results
(13) Pearson et al., 2010	Cross- sectional, ecological study conducted at the county level across the United States (U.S.)	General U.S. population; county-level (using data from over 3,000 counties) prevalence values of diagnosed diabetes for 2004 and 2005 created by the National Diabetes Surveillance System at the Centers for Disease Control and Prevention	10 µg/m³ increase in PM_2.5_ linked to 1% increase in diabetes prevalence in the U.S. (2004: β = 0.78 [95% CI 0.39 –1.25], p<0.001; 2005: β = 0.81 (95% CI: 0.48–1.07), p < 0.001)
(27) Kramer et al., 2010	Prospective cohort study	1,775 nondiabetic women from Germany, aged 54–55 at baseline	PM_2.5_ absorbance (soot) was positively associated with incident diabetes: HR 1.27 (1.09–1.48) per IQR, and 1.22 (1.02–1.47) among women with high C3c
(29) Chuang et al., 2011	Cross-sectional study	1,023 elderly participants (aged 54 and older) from Taiwan	Association between PM_2.5_ exposure and hyperglycemia, HbA1c, and inflammation markers in elderly subjects. For IQR increase in PM_2.5_, fasting glucose levels increased by 36.55 mg/dL (95% CI: 19.20 to 53.90)
(22) Kloog et al., 2012	Longitudinal prospective study	U.S. Medicare population, all residents aged 65 and older across New England (over 14 million residents)	Short-term PM_2.5_ exposure: every 10 µg/m³ increase was linked to a 0.96% increase in hospital admissions for diabetes (95% CI: 0.62–1.30). For every 10 µg/m³ increase in long-term PM_2.5_ exposure, hospital admissions for diabetes increased by 6.33% (95% CI: 3.22–4.59)
(35) Brook et al., 2013	Prospective cohort study	2.1 million Canadian adults	Each 10 µg/m³ increase in PM_2.5_ was associated with a 49% rise in mortality among individuals with diabetes with HR of 1.49 (95% CI 1.37–1.62)
(14) Chen et al., 2013	Prospective population-based cohort study	62,012 nondiabetic adults from Ontario, Canada	10 µg/m³ increase in PM_2.5_ associated with an 11% increased risk of diabetes (HR = 1.11, 95% CI: 1.02–1.21)
(34) Eze et al., 2014	Cross-sectional study	6,392 participants aged 29 to 73 from the Swiss Cohort Study on Air Pollution and Lung and Heart Diseases in Adults (SAPALDIA)	PM_10_ exposure linked to 40% increased diabetes risk (OR = 1.40, 95% CI: 1.17–1.67). The study did not model PM_2.5_, but due to the high correlation with PM_10_ (≈0.80) in SAPALDIA, the effects are expected to be essentially the same.
(36) Pope et al., 2015	Prospective cohort study	669,046 participants from the American Cancer Society Cancer Prevention Study II (ACS CPS-II)	Diabetes-related mortality associated with a 10 µg/m³ increase in PM_2.5_ exposure was 1.08 (95% CI: 0.97–1.19) when adjusted for individual-level covariates and increased to 1.13 (95% CI: 1.02–1.26) when ecological covariates were also included
(16) Weinmayr et al., 2015	Prospective cohort study	3,607 individuals from the Heinz Nixdorf Recall Study in Germany	PM_2.5_ exposure modestly increased diabetes incidence RR = 1.36 (95% CI: 0.98–1.89)
(17) Hansen et al., 2016	Longitudinal prospective cohort study	Danish Nurse Cohort with 28,731 female nurses	10 µg/m³ increase in PM_2.5_ linked to HR of 1.11 (95% CI: 1.02–1.22) for diabetes, especially in obese individuals in Denmark
(30) Liu et al., 2016	Cross-sectional study	Survey of 11,847 Chinese adults	41.1 µg/m³ increase in PM_2.5_ linked to a 14% increase in diabetes prevalence in China PR = 1.14 (95% CI: 1.08–1.20)
(37) Wolf et al., 2016	Cross-sectional study	2,944 participants from the KORA (Cooperative Health Research in the Region of Augsburg) F4 cohort, based in southern Germany	PM_2.5_ exposure was associated with a 9.7% increase in insulin resistance per 2.8 µg/m³ (95% CI: −1.3 to 21.9), rising to 26.8% among prediabetic individuals (95% CI: −0.8 to 62.1).
(15) Bowe et al., 2018	Longitudinal prospective cohort study	1,729,108 U.S. veterans	Each 10 µg/m³ increase in PM_2.5_ raised diabetes risk by 15% (HR) of 1.15 (95% CI: 1.08–1.22)
(28) Qiu et al., 2018	Prospective cohort study	61,447 elderly participants from Hong Kong	HR = 1.15 (95% CI: 1.05–1.25) per 3.2 µg/m³ increase in PM_2.5_ in elderly Hong Kong residents
(10) Lao et al., 2019	Longitudinal prospective cohort study	147,908 participants in Taiwan	Higher exposure to PM_2.5_ is associated with an increased risk of T2D, with a maximum HR of 1.28 (95% CI: 1.18–1.39) in the second exposure quartile compared to the lowest
(20) Li et al., 2019	Retrospective cohort study	505,151 participants in Taiwan	Each 10 µg/m³ increase in PM_2.5_ linked to 11% higher T2D incidence (HR = 1.11, 95% CI: 1.08–1.13) in Taiwan
(19) Chilian-Herrera et al., 2021	Cross-sectional study	2,275 adults aged 20 and older residing in the municipalities of the State of Mexico and Mexico City	10 µg/m³ increase in PM_2.5_ exposure associated with threefold diabetes risk increase (OR = 3.09, 95% CI: 1.17–8.15) in Mexico City
(21) Li et al., 2021	Prospective cohort study	449,006 participants from the U.K. Biobank	The highest level of PM_2.5_ exposure (fifth quintile) and T2D incidence was a 12% increased risk (HR: 1.12, 95% CI: 1.05–1.18) compared to the lowest quintile
(32) McAlexander et al., 2021	Prospective cohort study	U.S. suburban and rural adults, 11,208 participants from the REasons for Geographic and Racial Differences in Stroke (REGARDS) cohort	5 µg/m³ increase in PM_2.5_ exposure over two years was associated with increased odds of developing T2D. Suburban/small town communities: OR=1.65 (95% CI: 1.09–2.51). Rural communities: OR=1.56 (95% CI: 1.03–2.36)
(31) Laorattapong et al., 2023	Retrospective cohort study	21,325 Thai Army personnel	Higher PM_2.5_ exposure quartiles among Thai Army personnel linked to HRs for diabetes up to 2.40 (95% CI: 1.84–3.14)
(25) Li et al., 2023	Cross-sectional study	China Multi-Ethnic Cohort (CMEC), involving 69,210 adults aged 30 to 79 years from five provinces in Southwest China	8% higher odds of diabetes OR=1.08 (95% CI: 1.01–1.15) per-SD increase in PM_2.5_ concentration. Organic matter within PM_2.5_ had the strongest association, with an OR of 1.09 (95% CI: 1.02–1.16) per SD increase
(11) Liu et al., 2023	Prospective cohort study	It included 21,075 participants aged 45 and older from across China, China Health and Retirement Longitudinal Survey (CHARLS)	10 µg/m³ increase in PM_2.5_ linked to 26% increased diabetes risk (HR = 1.26, 95% CI: 1.22–1.31) in China
(12) Mandal et al., 2023	Prospective cohort study	Urban population in India. 2,064 adult participants from the cities of Chennai and Delhi, India	10 µg/m³ increase in PM_2.5_ linked to 1.22-fold diabetes risk increase (HR = 1.22, 95% CI: 1.09–1.36) in urban India
(18) Sørensen et al., 2023	Nationwide prospective cohort study	Denmark population, 1.8 million individuals aged 50–80	17% increased risk of T2D per 5 µg/m³ increase in PM_2.5_, with a HR=1.17 (95% CI: 1.13–1.21)
(24) Yitshak Sade et al., 2023	National prospective cohort study	41,780,637 individuals Medicare enrollees aged 65 and older in the contiguous U.S.	5 µg/m³ increase in PM_2.5_ linked to a 7.4% diabetes risk increase (HR = 1.074, 95% CI: 1.058–1.089)
(26) Cai et al., 2024	Cross-sectional study	Chinese adults. 129,244 participants from three regions in China (Fujian, Hubei, and Yunnan)	Interquartile range increase in PM_2.5_ associated with a 23% higher risk for diabesity (OR = 1.23, 95% CI: 1.17–1.30)
(33) Chung and Lin 2024	Nationwide prospective cohort study	158,038 Taiwanese adults	Higher PM_2.5_ quartiles linked to increased diabetes risk in Taiwan, with HR up to 1.42 (95% CI: 1.32–1.53) in the highest quartile
(23) Shen et al., 2024	Cohort prospective study	20,076,032 women age 20–49 years participating in the National Free Preconception Health Examination Project in China	Each interquartile range (27 μg/m^3^) increase in 3-year average PM_2.5_ concentration was associated with a 0.078 mmol/L (95% CI 0.077, 0.079) increase in FBG and 18% (95% CI 16%, 19%) higher risk of diabetes

C3c, complement component 3; HbA1c, glycated hemoglobin; RR, relative risk; HR, hazard ratio; OR, odds ratio; CI, confidence interval; IQR, interquartile range; SD, standard deviation.

#### Geographic distribution and exposure assessment

In Asia, where PM_2.5_ levels are among the highest globally, multiple studies have demonstrated significant associations. Lao et al. (10) reported that higher exposure to PM_2.5_ was associated with an increased risk of T2D, with a maximum hazard ratio (HR) of 1.28 (95% CI: 1.18–1.39) in the second exposure quartile compared to the lowest, in a Taiwanese population. Similarly, Liu et al. (11) observed a 26% increase in diabetes risk (HR = 1.26, 95% CI: 1.22–1.31) in China, and Mandal et al. (12) identified a 22% increase in risk (HR = 1.22, 95% CI: 1.09–1.36) per 10 µg/m³ increment in urban India. In North America, evidence from the United States (US) and Canada is robust. Pearson et al. (13) found a 1% increase in diabetes prevalence per 10 µg/m³ increase in PM_2.5_ across 3,000 U.S. counties (2004: β = 0.78 [95% CI: 0.39–1.25], p < 0.001; 2005: β = 0.81 [95% CI: 0.48–1.07], p < 0.001). In Ontario, Canada, Chen et al. (14) reported an 11% increased diabetes risk (HR = 1.11; 95% CI: 1.02–1.21) among 62,012 non diabetic adults, and Bowe et al. (15) demonstrated a 15% rise in diabetes risk for each 10 µg/m³ increase in PM_2.5_ (HR = 1.15; 95% CI: 1.08–1.22) among 1.7 million U.S. veterans. In Europe, several studies from Germany, Denmark, Switzerland, and the United Kingdom (UK) provide further support. In Germany, Weinmayr et al. (16) found a 36% increase in diabetes risk per 1 µg/m³ PM_2.5_ exposure, with a relative risk (RR) of 1.36 (95% CI: 0.97–1.89) among 3,607 participants. Hansen et al. observed an 11% increase in diabetes risk per 10 µg/m³ PM_2.5_ (HR = 1.11; 95% CI: 1.02–1.22) among Danish nurses (17), while Sørensen et al. (18) found a 17% increased risk per 5 µg/m³ PM_2.5_ in Denmark (HR = 1.17; 95% CI: 1.13–1.21), with higher risks among men and individuals with comorbidities. In other global regions, Chilian-Herrera et al. (19) reported from Mexico a threefold increase in diabetes risk per 10 µg/m³ PM_2.5_ exposure, with an odds ratio (OR) of 3.09 (95% CI: 1.17–8.15).

Average reported annual mean PM_2.5_ levels varied widely across study settings and time periods. In Taiwan, a two-year average PM_2.5_ concentration of 26.5 µg/m³ was reported (10). In mainland China, participants followed from 2011 to 2018 were exposed to an average city-level PM_2.5_ concentration of 54.3 µg/m³ (11). In India, median annual concentrations were 41.1 µg/m³ in Chennai and 92.1 µg/m³ in Delhi, two major urban centers (12). In the United States, county-level averages were approximately 10 µg/m³ during 2004–2005 (13), while nationwide data from 2003 to 2012 showed a range of 5.0 to 22.1 µg/m³ (15). In Canada, a six-year average (2001–2006) of 10.6 µg/m³ was observed, with higher values in southern Ontario (14). In Germany’s Ruhr area, average annual exposure between 2001 and 2002 was 16.7 µg/m³ (16). The Danish Nurse Cohort, covering 1995–2012, reported an average PM_2.5_ level of 18.1 µg/m³ (17), while a more recent Danish nationwide study (2005–2017) estimated a five-year time-weighted average of 10.9 µg/m³ (18). In the Mexico City Metropolitan Area, average annual PM_2.5_ concentrations ranged from 24 to 27 µg/m³ between 2004 and 2012 (19). Methods for estimating PM_2.5_ exposure varied widely across studies, ranging from ground-based monitoring to advanced satellite and chemical transport models, with spatial resolutions from neighborhood-level to several kilometers. These methodological differences affect exposure accuracy and likely contribute to heterogeneity in reported health associations.

#### Study design

The studies included in this review employ a variety of epidemiological designs to evaluate the association between PM_2.5_ exposure and the onset of T2D (**Table 1**). While prospective cohort studies (n=18) represent the most robust design for establishing causal relationships by following a population of healthy individuals over time, retrospective (n=2), cross-sectional studies (n=8) have also been included. The latter provide additional evidence supporting the association, although with some limitations in determining temporality and causality. The variety of study designs reflects the heterogeneous nature of the available literature on this topic. For instance, in Taiwan, Li et al. (20) using a retrospective design, reported an 11% increase in diabetes risk per 10 µg/m³ PM_2.5_ exposure (HR = 1.11; 95% CI: 1.08–1.13), particularly among older males and individuals with hyperlipidemia. Another study by Li et al. (21), using a prospective design, in the UK found that exposure to specific PM_2.5_ components, organic matter and black carbon, was associated with increased diabetes risk, showing 9% (HR = 1.09; 95% CI: 1.05–1.13) and 7% (HR = 1.07; 95% CI: 1.03–1.11) increases, respectively, per standard deviation. Several studies were conducted in large prospective cohorts, enhancing the robustness and generalizability of their findings. These include the U.S. Medicare cohort (22), which included about 11 million hospital admissions among Medicare beneficiaries in New England; the Chinese National Free Preconception Health Examination Project (23), comprising 20,076,032 women aged 20–49; and a national U.S. cohort (24) of 41,780,637 older adults enrolled in Medicare. Eight cross-sectional studies also provided valuable insights into exposure-response relationships, though they are more limited in establishing causality. In China, Li et al. (25) reported that each standard deviation increases in the three-year average PM_2.5_ concentration was associated with an 8% increase in the odds of diabetes based on fasting blood glucose (FBG) levels among adults without prior diabetes (OR = 1.08; 95% CI: 1.01–1.15). Cai et al. (26) found a 23% increased diabetes risk per interquartile range increase in PM_2.5_ exposure in a multicenter Chinese cohort (OR = 1.23; 95% CI: 1.17–1.30), with organic matter (48%) and black carbon (30%) identified as key chemical contributors.

#### Population characteristics

Population subgroups may differ in their susceptibility to air pollution. Women appear particularly vulnerable to PM_2.5_ exposure, as shown by elevated diabetes risk in Danish nurses (17). For instance, German women with high circulating levels of complement factor C3c, a marker of subclinical inflammation, were found to have an increased risk of developing T2D in relation to traffic-related air pollution exposure (27). Similarly, in a large cohort of Chinese women of reproductive age, higher long-term exposure to PM_2.5_ was associated with elevated fasting plasma glucose and an increased risk of impaired fasting glucose, suggesting enhanced metabolic susceptibility in this demographic group (23). Elderly individuals are another high-risk group. In Hong Kong, a 15% increase in diabetes risk (HR = 1.15; 95% CI: 1.05–1.25) was observed per 3.2 µg/m³ PM_2.5_ among older adults (28). In Taiwan, associations were found between PM_2.5_ levels and markers such as hyperglycemia, HbA1c, and systemic inflammation in elderly populations (29). In China, a 14% increase in diabetes incidence (prevalence ratio [PR] = 1.14; 95% CI: 1.08–1.20) associated with PM_2.5_ exposure was more pronounced in men, smokers, the elderly, and obese individuals (30). Obesity emerged as a consistent susceptibility factor across multiple studies in Denmark, China, and the U.K (17, 18, 21, 26, 30), reinforcing the idea that metabolic comorbidities may heighten the effects of air pollution.

#### Key outcomes

The risk estimates across the studies were consistently positive, with odds ratios ranging from modest (OR = 1.01) to substantial (OR = 3.09). Greater increases in PM_2.5_ exposure were typically associated with steeper increases in diabetes risk. In Mexico, Chilian-Herrera et al. reported a threefold increase in diabetes risk (OR = 3.09; 95% CI: 1.17–8.15) per 10 µg/m³ PM_2.5_ (19). Laorattapong et al. found a 2.40-fold increase in risk (HR = 2.40; 95% CI: 1.84–3.14) for individuals in the highest quartile of PM_2.5_ exposure (31). McAlexander et al. (32) observed that a 5 µg/m³ increase in PM_2.5_ over two years was associated with a 65% increase in T2D odds in suburban/small-town communities (OR=1.65; 95% CI: 1.09–2.51) and a 56% increase in rural areas of the U.S (OR=1.56; 95% CI: 1.03–2.36). In Taiwan, Chung and Lin (33) reported a 1.42-fold higher risk of diabetes in the highest PM_2.5_ quartile (HR=1.42; 95% CI: 1.32–1.53), while in Switzerland, Eze et al. (34) found a 40% increased diabetes risk per 10 µg/m³ PM_10_ exposure (OR = 1.40, 95% CI: 1.17–1.67). This study did not model PM_2.5_, but the authors note that PM_2.5_ and PM_10_ are highly correlated in SAPALDIA (PM_2.5_/PM_10_ ≈ 0.80), so the PM_10_ effects would be essentially the same for PM_2.5_. Regarding mortality and severe outcomes, significant associations were noted. Brook et al. (35) reported a 49% increase in diabetes-related mortality with higher long-term PM_2.5_ exposure (HR = 1.49; 95% CI: 1.37–1.62), while Pope et al. (36) found a 13% increase in mortality risk linked to PM_2.5_ (HR = 1.13; 95% CI: 1.02–1.26). Pathophysiological effects were also consistently reported. Elevated fasting glucose, HbA1c levels, and inflammatory biomarkers were associated with increased PM_2.5_ exposure. For instance, Chuang et al. (29) observed a 36.55mg/dL increase in fasting glucose (95% CI: 19.20 to 53.90) per interquartile range increase in PM_2.5_. Wolf et al. (37) observed a 9.7% increase in insulin resistance (HOMA-IR) per 2.8 µg/m³ increase in PM_2.5_ (95% CI: −1.3 to 21.9), rising to 26.8% among prediabetic individuals (95% CI: −0.8 to 62.1).

#### Studies reporting weak or null associations between PM_2.5_ exposure and T2D

Although most studies support a positive association between PM_2.5_ exposure and T2D, a few have reported weak or null findings, as summarized in **Table 2**. These studies highlight heterogeneity in results that may be due to differences in study design, exposure assessment, population characteristics, or regional pollution profiles. A prospective cohort study (38) involving 4,204 African American women in Los Angeles from the Black Women’s Health Study (BWHS) found no significant association between 10 µg/m³ increases in PM_2.5_ and T2D incidence. Similarly, Puett et al. (39) analyzed two large U.S. cohorts comprising 74,412 women and 15,048 men and reported no significant association between PM_2.5_ exposure in the 12 months prior to diagnosis and the onset of diabetes. However, an association emerged among women when exposure was defined by residential proximity to high-traffic roads, suggesting that localized pollution metrics may reveal associations not captured by broader models. In the Netherlands, Dijkema et al. (40) conducted a cross-sectional study among 8,018 adults aged 50–75 years and found no consistent relationship between long-term traffic-related air pollution and T2D prevalence. Although small increases in T2D incidence were noted near high-traffic areas and a slight, non-significant trend suggested greater susceptibility among women, the overall findings did not indicate a clear exposure–response relationship. In peri-urban South India, Curto et al. (41) conducted a cross-sectional study of 5,065 adults from 28 villages and found no significant associations between residential exposure to PM_2.5_ or black carbon and FBG levels or diabetes status. While personal exposure to PM_2.5_ and black carbon was negatively associated with blood glucose levels in women, the overall results were consistent with the null hypothesis and did not support a link between these pollutants and diabetes outcomes. These studies, although fewer in number, underscore the need for more refined exposure assessment techniques and consideration of population-specific factors to better understand potential inconsistencies in the observed associations.

**Table 2:** Principal studies against the association between PM_2.5_ exposure and T2D.

Study/reference	Study type	Population type	Results
(40) Dijkema et al., 2011	Cross-sectional study	8,018 participants aged 50 to 75 years from a semirural region in the Netherlands	No consistent association was found between long-term traffic-related air pollution and T2D prevalence
(39) Puett et al., 2011	Prospective cohort study	U.S. 74,412 women, and 15,048 men	No significant association between PM_2.5_ or PM_10_ exposure and diabetes incidence
(38) Coogan et al., 2012	Prospective cohort study	4,204 black women living in Los Angeles, drawn from the Black Women’s Health Study (BWHS)	No significant association between a 10 µg/m³ PM_2.5_ increase and T2D
(41) Curto et al., 2019	Cross-sectional study	5,065 adults from 28 peri-urban villages near Hyderabad, South India	No significant association between PM_2.5_ or black carbon exposure and blood glucose or diabetes status

#### Systematic reviews and meta-analyses on the association between PM_2.5_ exposure and T2D

Several systematic reviews and meta-analyses have investigated the potential causal role of PM_2.5_ exposure in the development of T2D, providing a broader perspective on the overall body of evidence. These reviews, summarized in **Table 3**, generally support a modest but consistent association between exposure to PM_2.5_, and increased T2D risk. Balti et al. (42) in a meta-analysis including ten studies from the U.S., Canada, and Europe, found a statistically significant association between PM_2.5_ exposure and an 11% increase in T2D risk per 10 µg/m³ (RR = 1.11, 95% CI: 1.03–1.19). Li et al. (43) analyzed 12 studies and reported that PM_2.5_ exposure was associated with a 12.3% increase in diabetes mortality (RR = 1.12, 95% CI: 1.03–1.21). Similarly, Park and Wang (44) reviewed 22 studies and found a 10% increase in T2D risk per 10 µg/m³ PM_2.5_ (pooled RR = 1.10, 95% CI: 1.03–1.18). Other meta-analyses yielded comparable findings. Wang et al. (45) in their analysis of ten cohort studies involving 2.37 million participants, reported that PM_2.5_ exposure increased diabetes risk by 39% per 10 µg/m³ (RR = 1.39; 95% CI: 1.14–1.68), and by 28% in categorical comparisons (RR = 1.28; 95% CI: 1.06–1.55). Eze et al. (46) included 13 studies and observed a 10% increase in T2D risk per 10 µg/m³ of PM_2.5_ (RR = 1.10, 95% CI: 1.02–1.18), with a higher risk observed among women (RR = 1.14; 95% CI: 1.03–1.26). Janghorbani et al. (47) who reviewed 17 studies involving over 4 million participants, found a significant association for PM_10_ (1.008; 95% CI: 1.003-1.013), but not for PM_2.5_ (OR = 1.05, 95% CI: 0.99–1.10). Dendup et al. (48) analyzing 60 studies, concluded that air pollution is a moderate risk factor for T2D with a pooled OR of 1.09 (95% CI: 1.04–1.15) for each incremental increase in PM_2.5_ exposure, though they noted substantial heterogeneity across study quality and populations. Yang et al. (49) reviewed 86 studies and confirmed significant associations between long-term exposure to PM_2.5_ and the prevalence and incidence of T2D, with pooled effect estimates ranging from OR = 1.08 (95% CI: 1.04–1.12) to HR = 1.11 (95% CI: 1.00–1.22), respectively, per 10 µg/m³ increase. Finally, Kutlar Joss et al. (50) evaluated 21 studies on traffic-related air pollution and found that each 1 µg/m³ increase in PM_2.5_ was associated with a 3% higher likelihood of T2D (OR = 1.03, 95% CI: 1.01–1.05). Taken together, these meta-analyses support the hypothesis that PM_2.5_ exposure is associated with a modest but measurable increase in the risk of T2D, although the strength of the association varies depending on pollutant type, exposure assessment, and study population.

**Table 3:** Principal meta-analyses and systematic reviews on PM_2.5_ exposure and T2D.

Study/reference	Study type	Studies (n°)	Results
(42) Balti et al., 2014	Systematic review and meta-analysis	10	Increased T2D risk (HR = 1.11, 95%CI, 1.03-1.20; p < 0.001; I (2) = 0.0%, pheterogeneity = 0.827) per 10 µg/m³ increase in PM_2.5_ across cohorts
(43) Li et al., 2014	Systematic review and meta-analysis	12	PM_2.5_: a 10 μg/m³ increase in PM_2.5_ was associated with a 12.3% increase in diabetes-related mortality risk (RR = 1.12, 95% CI: 1.03–1.21).
(47) Janghorbani et al., 2014	Systematic review and meta-analysis	17	OR = 1.008 (95% CI: 1.003-1.013) per 10 µg/m³ increase in PM_10_ for diabetes; no significant association for PM_2.5_ (OR = 1.05 (95% CI: 0.99-1.10)
(44) Park and Wang 2014	Systematic review	22	PM_2.5_: For each 10 µg/m³ increase in PM_2.5_ exposure, there was an associated 10% increase in the risk of developing T2D (pooled RR = 1.10, 95% CI: 1.03–1.18).
(45) Wang et al., 2014	Systematic review and meta-analysis	10	PM_2.5_ exposure increased diabetes risk by 39% per 10 µg/m³ (RR = 1.39; 95% CI: 1.14–1.68), and by 28% in categorical comparisons (RR = 1.28; 95% CI: 1.06–1.55).
(46) Eze et al., 2015	Systematic review and meta-analysis	13	10% increased diabetes risk per 10 µg/m³ increase in PM_2.5_ 1.10 (95% CI: 1.02, 1.18)
(48) Dendup et al., 2018	Systematic review	60	PM_2.5_ was identified as a risk factor for T2D: each 10 µg/m³ increase in long-term PM_2.5_ exposure was linked to an 11% higher diabetes incidence (HR = 1.11; 95% CI: 1.02–1.21).
(49) Yang et al., 2020	Systematic review and meta-analysis	86	Significant associations of PM_2.5_ with T2D incidence (11 studies; HR = 1.10, 95% CI = 1.04-1.17 per 10 μg/m^3^ increment; I^2^ = 74.4%) and prevalence (11 studies; OR = 1.08; 95% CI = 1.04-1.12 per 10 μg/m^3^ increment; I^2^ = 84.3%)
(50) Kutlar Joss et al., 2023	Meta-analysis	21	OR = 1.03 (95% CI: 1.01–1.05) per 1 µg/m³ increase in PM_2.5_ for diabetes prevalence

HbA1c, glycated hemoglobin; RR, relative risk; HR, hazard ratio; OR, odds ratio; CI, confidence interval.

In summary, epidemiological studies on causal associations between prolonged PM_2.5_ exposure, and the onset of T2D have produced inconsistent and hardly comparable results. These differences may be explained by the imprecision in assessing exposure to both PM_2.5_ and other atmospheric pollutants. Indeed, variations in exposure levels and study methodologies may account for the observed consistencies and discrepancies in the findings (51).

### Short-term exposure

Short-term exposure to ambient PM_2.5_, has been linked to alterations in glucose metabolism and increased diabetes-related mortality, as highlighted in **Table 4**. Short-term exposure refers to periods ranging from approximately one week up to one month prior to the measurement of health biomarkers or parameters. Zhan et al. (52) conducted a longitudinal study of 47,471 individuals in Eastern China and found that a 10 µg/m³ increase in PM_2.5_ was associated with significant elevations in FBG at multiple lag times. In single-pollutant models, increases ranged from 0.0030 mmol/L (lag 0–7 days; 95% CI: –0.0005 to 0.0066; *ns*) to 0.0325 mmol/L (lag 0–28 days; 95% CI: 0.0261 to 0.0390; *p* < 0.001). In multipollutant models, the effect was even stronger, reaching 0.0387 mmol/L (95% CI: 0.0317 to 0.0456; *p* < 0.001) for a 29-day moving average, indicating that short-term PM_2.5_ exposure may meaningfully influence glucose homeostasis. Peng et al. (53) studied a panel of 70 healthy adults and reported that each 10 µg/m³ increase in 3-day moving average PM_2.5_ was associated with a 4.19% increase in fasting insulin (95% CI: 2.35–6.05), a 5.95% increase in β-cell function (HOMA-B; 95% CI: 3.66–8.26), and a 3.77% increase in insulin resistance (HOMA-IR; 95% CI: 1.41–4.64). No significant change in FBG was observed, but effects were more pronounced among overweight individuals and those with low physical activity levels. Brook et al. (54) investigated short-term PM_2.5_ exposure (4–5 hours/day for 5 days) in 25 healthy individuals and found a positive correlation with insulin resistance (HOMA-IR +0.7; 95% CI: 0.1–1.3; *p* = 0.023) and a negative correlation with heart rate variability (HRV), particularly SDNN (–13.1 ms; 95% CI: –25.0 to –1.2; *p* = 0.032) per 10 µg/m³ PM_2.5_ increase. These findings suggest that short-term exposure to PM_2.5_ can disrupt glucose metabolism, promote insulin resistance, and increase the risk of diabetes-related mortality, effects that may be particularly relevant in vulnerable populations.

**Table 4:** Short-term effects of PM_2.5_ exposure on glucose regulation and diabetes outcomes.

Study	Population	Exposure	Findings	Key implications
(54) Brook et al., 2013	25 healthy individuals	PM_2.5_ (4–5 hours/day)	HOMA-IR: +0.7. HRV (SDNN): -13.1 ms per 10 μg/m³ increase in PM_2.5_	Short-term PM_2.5_ exposure linked to metabolic dysregulation, indicating potential causal links to T2D development
(52) Zhan et al., 2021	47,471 individuals in Eastern China	PM_2.5_ (10 μg/m³)	FBG increases: 0.0030 mmol/L (lag 0–7 days), 0.0233 mmol/L (lag 0–21 days), 0.0325 mmol/L (lag 0–28 days). Multipollutant models: 0.0387 mmol/L (29-day avg)	Short-term PM_2.5_ exposure significantly increases FBG levels, suggesting higher diabetes risk
(53) Peng et al., 2022	70 healthy adults	PM_2.5_ (10 μg/m³)	Fasting insulin: +4.19%. HOMA-B: +5.95%. HOMA-IR: +3.77%. No significant FBG change	More pronounced effects in overweight individuals and those with low physical activity

FBG, fasting blood glucose; HOMA-IR, homeostasis model assessment of insulin resistance; HOMA-B, homeostasis model assessment of β-cell function; lag, lag time; CI, confidence interval; SDNN, standard deviation of normal-to-normal intervals.

### Experimental animal studies

Experimental studies in animal models have provided mechanistic support for the epidemiological associations between PM_2.5_ exposure and T2D, as summarized in **Table 5**. These studies demonstrate how PM_2.5_ can affect insulin sensitivity, glucose metabolism, and systemic inflammation. Xu et al. (55), showed that chronic exposure to PM_2.5_ in C57BL/6J wild-type male mice induced glucose intolerance and increased HOMA-IR, indicating reduced insulin sensitivity. In a related study, Xu et al. (56) found that acute PM_2.5_ exposure initiated at 3 weeks of age induced insulin resistance and inflammation in C57BL/6J wild-type male mice, effects that were attenuated in NADPH oxidase p47phox-knockout mice, suggesting a role for oxidative stress in mediating these effects. Laing et al. (57) demonstrated that PM_2.5_ exposure induces endoplasmic reticulum (ER) stress in lung and liver tissues via activation of the unfolded protein response (UPR), particularly the PERK-eIF2α-CHOP pathway, leading to cellular apoptosis. This study was conducted in male C57BL/6J mice. Given the liver’s central role in glucose and lipid metabolism, ER stress in this organ can impair insulin signaling pathways and contribute to hepatic insulin resistance, a key feature in the pathogenesis of T2D. Liu et al. (58) found that genetically diabetic male mice (KKay strain) exposed to PM_2.5_ for 5–8 weeks exhibited impaired energy metabolism, increased visceral adiposity, and worsened insulin resistance. This was accompanied by dysregulation of key adipokines, including reduced adiponectin and increased leptin, and a reduction in thermogenic gene expression in brown adipose tissue. Animal studies also indicate a role for PM_2.5_ in promoting obesity in both young (56) and adult mice, showing that 10 weeks of high PM_2.5_ exposure exacerbates obesity-induced insulin resistance and visceral inflammation (59) in adult male C57BL/6J mice. Consistent with these observations, impaired hepatic insulin signaling in response to PM_2.5_ exposure was evidenced by increased insulin receptor substrate 1 (IRS-1) serine phosphorylation at sites 1101 and 636 in the liver after 10 weeks of PM_2.5_ exposure (60). This was demonstrated in male C57BL/6J mice. Some researchers also suggest that PM_2.5_ may contribute to the pathogenesis of T2D through the central nervous system by inducing hypothalamic inflammation, which could disrupt metabolic regulation and influence insulin resistance, potentially via alterations in sympathetic nervous system (SNS) activity (6). This study was conducted in adult male Swiss Webster mice. Collectively, these animal studies confirm that PM_2.5_ can directly impair metabolic pathways and support the biological plausibility of its role in T2D pathogenesis. Additional experimental evidence in rats supports the hypothesis that PM_2.5_ exposure contributes to cardiovascular and metabolic dysfunction relevant to diabetes pathogenesis. In a study by Carll et al. (61), Sprague Dawley rats with metabolic syndrome induced by a high-fructose diet were exposed to ambient-level traffic-derived PM_2.5_ (20.4 ± 0.9 μg/m³) for 12 days. This exposure significantly decreased HRV and baroreflex sensitivity, shortened ECG intervals (PR and QTc), and increased arrhythmia incidence, particularly atrioventricular block type II, suggesting autonomic imbalance and heightened cardiovascular vulnerability in metabolically compromised rats. In a separate study, Long et al. (62) demonstrated that PM_2.5_ exacerbates glucose intolerance in Zucker rats through interleukin-6 (IL-6)–mediated systemic inflammation, reinforcing a mechanistic link between air pollution, inflammation, and diabetes onset. Similarly, Yan et al. (63) reported that subchronic inhalation of ambient PM_2.5_ in streptozotocin-induced diabetic rats worsened glucose homeostasis and led to organ damage, particularly in the pancreas and kidney.

**Table 5:** Experimental animal studies.

Study	Model/system	Exposure/findings	Key implications
(59) Sun et al., 2009	Adult male C57BL/6J mice	10 weeks of high PM_2.5_ exposure exacerbated obesity-induced insulin resistance and visceral inflammation	Supports PM_2.5_’s role in worsening obesity-related metabolic dysfunction
(56) Xu et al., 2010	Wild-type and NADPH oxidase P47phox-/- knockout young male C57BL/6J mice	Chronic PM_2.5_ exposure initiated at 3 weeks of age caused insulin resistance and inflammation. Effects were attenuated in knockout mice, suggesting NADPH oxidase mediation	Identifies NADPH oxidase as a mediator of PM_2.5_-induced metabolic effects. Highlights PM_2.5_’s role in inducing obesity and related metabolic disturbances
(57) Laing et al., 2010	C57BL/6J male mice	PM_2.5_ exposure induced ER stress in lung and liver tissues via the PERK-eIF2α-CHOP pathway, leading to apoptosis	Reveals molecular mechanisms linking PM_2.5_ exposure to cytotoxicity and pollution-related diseases
(55) Xu et al., 2011	C57BL/6J wild-type male mice	ChronicPM_2.5_ exposure caused glucose intolerance and increased HOMA-IR, indicating reduced insulin sensitivity	Demonstrates the role of PM_2.5_ in impairing glucose metabolism and inducing insulin resistance
(60) Zheng et al., 2013	Male C57BL/6J mice	PM_2.5_ exposure for 10 weeks increased hepatic IRS-1 serine phosphorylation, impairing hepatic insulin signaling	Provides evidence of disrupted hepatic insulin signaling due to PM_2.5_ exposure
(58) Liu et al., 2014	KKay diabetic male mice	5–8 weeks of PM_2.5_ exposure impaired energy metabolism, increased visceral adiposity, and worsened insulin resistance. Adipokine dysregulation was observed, with reduced adiponectin and increased leptin levels	Highlights PM_2.5_’s role in exacerbating metabolic dysfunction in genetically susceptible diabetic mice
(76) Liu et al., 2014	C57BL/6 mice	Concentrated PM_2.5_ for several weeks	Reductions in HRV, increased sympathetic drive, and elevated norepinephrine levels
(63) Yan et al., 2014	Streptozotocin-induced diabetic rats (male Wistar)	Inhaled ambient PM_2.5_ For 4 h/day, 5 days/week, for 4 weeks	Increased blood glucose, kidney and pancreatic oxidative stress and inflammation, impaired glucose tolerance. Mechanisms: Increased TNF-α expression, oxidative tissue damage
(101) Ying et al., 2014	Male C57BL/6J mice	PM_2.5_ exposure activated the sympathetic nervous system, inducing insulin resistance via central nervous system pathways	Suggests CNS-mediated mechanisms for PM_2.5_-induced T2D pathogenesis
(61) Carll et al., 2017	Male Sprague-Dawley rats, fructose-fed (metabolic syndrome model)	Inhaled ambient-level traffic-derived PM_2.5_ (20.4 ± 0.9 μg/m³), 6 h/day for 12 consecutive days	Decreased HRV, baroreflex sensitivity, increased arrhythmias (esp. AV block type II), shortened PR and QTc intervals
(62) Long et al., 2020	Male Zucker diabetic fatty (ZDF) rats	Whole-body inhalation of concentrated ambient PM_2.5_ for 8 h/day for 8 weeks	Increased fasting glucose and insulin, increased HOMA-IR and liver inflammation through IL-6–mediated activation of STAT3/SOCS3 pathway in liver, systemic inflammation
(6) Campolim et al., 2020	C57BL/6J mice (6–10 weeks old), Swiss Webster mice	Acute PM_2.5_ exposure (1–5 days): no effect after 1 day; after 5 days, increased hypothalamic inflammation, fat mass, food intake. Chronic PM_2.5_ exposure (12 weeks): obesity, hyperphagia, insulin and leptin resistance	Trigger of hypothalamic inflammation independently of diet, leading to metabolic disorders

HOMA-IR, homeostasis model assessment of insulin resistance; NADPH, nicotinamide adenine nucleotide phosphate (reduced form); ER, endoplasmic reticulum; IRS-1, insulin receptor substrate-1; CNS, central nervous system; AV, atrioventricular; PR interval, atrioventricular conduction interval; QTc, corrected QT interval; IL-6, interleukin-6; STAT3/SOCS3 Pathway, Signal Transducer and Activator of Transcription 3/Suppressor of Cytokine Signaling 3.

Based on the evidence reviewed above, long-term and short-term epidemiological studies consistently link PM_2.5_ exposure to markers of glucose dysregulation, while experimental animal models corroborate these findings by demonstrating mechanistic pathways, such as adipose inflammation and hypothalamic leptin resistance, under controlled exposure regimens. Together, these data emphasize the concordance between population-based observations and preclinical mechanistic insights regarding PM_2.5_-induced metabolic dysfunction.

### Emerging mechanisms and nervous-system involvement

**Figure 2:**
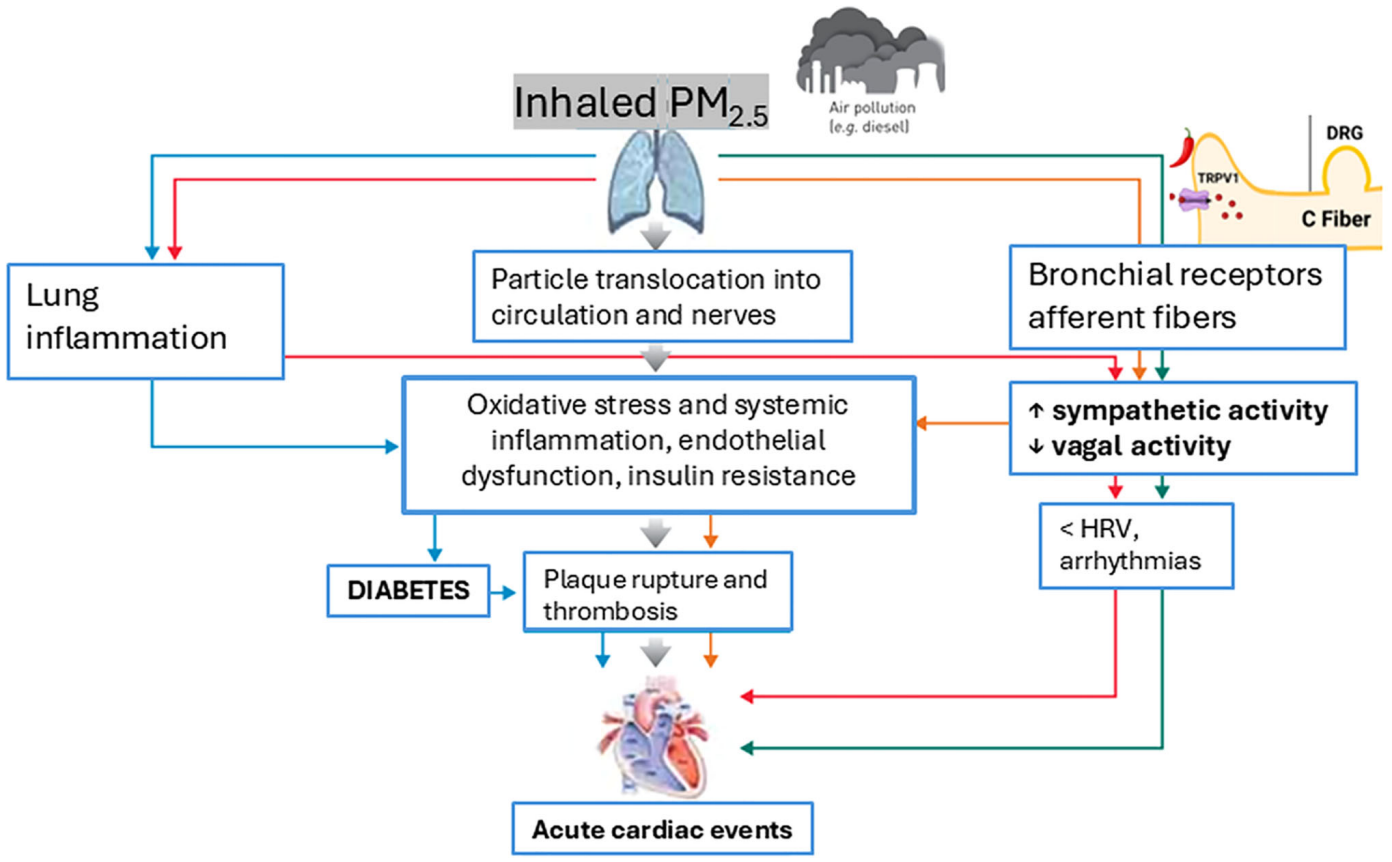
Role of PM_2.5_ exposure in the development of T2D and related acute cardiac events through key mechanisms. Adapted from Lottti (102).

Air pollution contributes to the development of T2D through multiple interconnected mechanisms (**Figure 2**). These pathways include systemic and lung inflammation, oxidative stress, adipose tissue inflammation, autonomic nervous system dysregulation and endothelial dysfunction. Together, these mechanisms provide a framework for understanding how PM_2.5_ exposure can disrupt glucose metabolism and exacerbate insulin resistance.

### Oxidative stress and systemic inflammation

Evidence from human studies supports a key role for oxidative stress in mediating the metabolic effects of air pollution. PM_2.5_ generates reactive oxygen species (ROS) that damage lipids, proteins, and DNA, leading to systemic inflammation. This inflammatory response is marked by elevated levels of cytokines such as IL-6, tumor necrosis factor-aplha (TNF-α), and C-reactive protein, which can impair insulin signaling pathways and contribute to insulin resistance (4). These proinflammatory, prothrombotic, and oxidative effects are among the most consistently observed mechanisms linking PM_2.5_ exposure to the development of cardiovascular and metabolic diseases (64). Short-term human studies, although variable in results, also suggest a role for inflammation. For instance, O’Neil et al. (65) reported associations between PM_2.5_ exposure over the previous six days and increased levels of inflammatory markers, including soluble intercellular adhesion molecule-1 (sICAM-1), vascular cell adhesion molecule-1 (VCAM-1), and von Willebrand factor, in individuals with diabetes, although the associations did not reach statistical significance.

Findings from animal studies further support these mechanisms. Controlled exposure to PM_2.5_ in rodents has been shown to induce systemic inflammation and oxidative stress, with downstream effects on insulin sensitivity. Xu et al. (55) demonstrated that long-term PM_2.5_ exposure in wild-type mice caused glucose intolerance and increased HOMA-IR, consistent with reduced insulin sensitivity, which was accompanied by adipose tissue inflammation and oxidative stress, supporting a mechanistic link between PM_2.5_ and metabolic dysfunction. In a related study, Xu et al. (56) showed that short-term exposure initiated at 3 weeks of age induced insulin resistance and inflammation in wild-type mice, effects that were attenuated in NADPH oxidase p47phox knockout mice, suggesting a central role for oxidative stress in mediating these outcomes.

### Adipose tissue inflammation and insulin resistance

Pollutants also target adipose tissue, inducing localized inflammation that disrupts lipid metabolism and exacerbates insulin resistance. This mechanism has been demonstrated in animal studies. PM_2.5_ exposure has been linked to increased visceral adiposity and altered adipokine levels, including reduced adiponectin and elevated leptin, both of which are critical regulators of glucose metabolism (58, 59). These adipose tissue alterations are accompanied by macrophage infiltration and increased expression of proinflammatory cytokines, such as TNF-α and IL-6, which interfere with insulin signaling pathways, particularly by promoting serine phosphorylation of insulin receptor substrates. This cascade impairs glucose uptake and contributes to systemic insulin resistance, a hallmark of T2D pathogenesis (66). Although most of the mechanistic data derive from animal models, human-based reviews support a similar role for adipose tissue–derived inflammation in metabolic dysfunction (67). Furthermore, a randomized, controlled, crossover human exposure study have linked PM_2.5_ exposure, even at low levels, to markers of insulin resistance and low-grade systemic inflammation (54).

#### Dysregulation of the autonomic nervous system

Another hypothesized mechanism involves the autonomic nervous system. Exposure to PM_2.5_ has been shown to induce rapid autonomic imbalance, as evidenced by reduced HRV (54). This imbalance can result in altered insulin sensitivity and peripheral inflammation (68), potentially explaining the consistent associations among reduced HRV, glucose intolerance, and inflammation (69, 70). Furthermore, autonomic imbalance characterized by heightened sympathetic activity can disrupt glucose metabolism by impairing insulin sensitivity (71–73). Supporting this, Rankin et al. (74) demonstrated that short-term exposure to diesel exhaust particles (DEPs), a significant source of PM_2.5_ (75), rapidly increases muscle sympathetic nerve activity (MSNA) in humans. Participants inhaled for 40 min DEPs adjusted to a particulate mass concentration of 304 ± 7 µg/m³ with a size distribution indicating a predominance of fine/ultrafine particles and co-pollutants of total hydrocarbons 0.78 ± 0.05 ppm, NOx 3.42 ± 0.38 ppm (nitrogen dioxide 0.02 ppm). The increase in MSNA occurred within 10 minutes of exposure and peaked at 30 minutes, correlating with autonomic imbalance and suggesting a plausible pathway linking particulate exposure to metabolic and cardiovascular effects. In addition, animal studies have shown that PM_2.5_ exposure increases sympathetic outflow and impairs autonomic regulation. For example, Carll et al. (61) reported that spontaneously hypertensive rats exposed to concentrated ambient particles exhibited significant elevations in sympathetic nerve activity and arterial pressure. Similarly, Liu et al. (76) demonstrated that C57BL/6 mice exposed to concentrated PM_2.5_ for 6 months developed reduced HRV and enhanced sympathetic tone, further supporting the role of pollution-induced autonomic dysregulation. Collectively, human and experimental studies converge on a model in which PM_2.5_ exposure impairs autonomic balance and metabolic regulation via sympathetic overactivity. These observations are consistent with the framework proposed by Rajagopalan et al. (4), who note that acute reductions in HRV, increases in blood pressure and catecholamines after PM_2.5_/ultrafine exposure, and the link between sympathetic activation and insulin resistance, support sympathetic and likely Hypothalamic–Pituitary–Adrenal-axis pathways as key mediators of cardiometabolic dysfunction.

#### Endothelial dysfunction

Endothelial dysfunction induced by PM_2.5_ exposure impairs the vascular transport of glucose into tissues, further contributing to hyperglycemia and insulin resistance. The disruption of endothelial integrity is linked to increased serum intercellular adhesion molecules and vascular cell adhesion molecules, as observed in the study by Madrigano et al. (77), where a pronounced effect was observed in obese subjects exposed to carbon black rather than PM_2.5_. Recent evidence (4) confirm endothelial barrier disruption via oxidative stress, nuclear factor kappa-light-chain-enhancer of activated B cells (NF-κB) activation and increased ICAM-1/VCAM-1 as a central pathway through which PM_2.5_ blunts insulin-mediated vasodilation and microvascular glucose delivery. This vascular bottleneck plausibly links PM_2.5_ exposure to systemic insulin resistance and broader cardiometabolic risk. Consistent with these human-based findings, Sun et al. (59) demonstrated that chronic PM_2.5_ exposure in C57BL/6 mice induced clear signs of endothelial dysfunction. Mice exposed for 24 weeks to concentrated ambient PM_2.5_ exhibited impaired acetylcholine- and insulin-mediated vasodilation, reduced nitric oxide bioavailability, and downregulation of endothelial nitric oxide synthase (eNOS) expression in the aortic endothelium. These vascular changes were accompanied by increased oxidative stress and reduced phosphorylation of protein kinase B (Akt), supporting a direct link between PM_2.5_ exposure and vascular insulin resistance.

#### Remaining gaps and consistencies

Despite these advances, inconsistencies remain in the mechanistic understanding of the effects of PM_2.5_ on diabetic individuals. Factors such as variations in exposure levels, pollutant composition, and study methodologies may contribute to discrepancies in findings. More specifically, key uncertainties persist regarding how oxidative signaling evolves from short-term to long-term exposure, the concentration–duration thresholds that sustain autonomic dysregulation, the extent to which endothelial dysfunction actually limits insulin’s microvascular delivery to muscle and adipose tissue *in vivo* and the absence of integrated multi-omic datasets that map convergent pathways in the same exposed subjects. For example, the relationship between reduced HRV and glucose intolerance remains incompletely understood but is likely multifactorial, involving autonomic imbalance, systemic inflammation, and oxidative stress. While individual studies provide partial insights, the cumulative evidence supports a plausible causal association between PM_2.5_ exposure and T2D. Mechanistic pathways such as oxidative stress, inflammation, and autonomic dysregulation offer a comprehensive framework for understanding how PM_2.5_ exposure exacerbates metabolic disorders. These findings underscore the importance of reducing PM_2.5_ exposure to mitigate the global burden of T2D.

### Role of transient receptor potential vanilloid 1 in the link between PM_2.5_ exposure and T2D

Transient receptor potential vanilloid 1 (TRPV1) is a nonselective cationic ligand-gated channel with high permeability to calcium ions (Ca²^+^). It is expressed on capsaicin-sensitive sensory neurons and various non-neuronal cell membranes, including inflammatory cells (e.g., macrophages and T lymphocytes), skeletal muscle, and brain nuclei, albeit at lower levels (78–81). These TRPV1-expressing sensory neurons innervate key metabolic organs, such as the pancreas and adipose tissue (82–84), and TRPV1 has also been detected in pancreatic β cells (85) and adipocytes (86), suggesting its potential role in glucose metabolism.

#### TRPV1 and metabolic regulation

TRPV1 plays a dual role in pain and metabolic regulation. It mediates pancreatitis-associated pain and influences glucose metabolism. TRPV1 channels regulate insulin secretion in pancreatic β cells through calcium-dependent mechanisms, while their activation in adipocytes enhances thermogenesis and energy expenditure, contributing to systemic metabolic homeostasis (87). Additionally, TRPV1 expression in the hypothalamus has been linked to appetite regulation and glucose metabolism via central signaling pathways (87). Studies in diabetic rodent models, including streptozotocin-induced diabetic rats, obese Zucker rats, and high-fat diet-fed mice, have shown that TRPV1 ablation improves glucose tolerance, reduces obesity, and highlights its potential as a therapeutic target for diabetes and weight management (88). TRPV1-positive neurons release neuropeptides, such as SP, IL-6, and CGRP, which are critical mediators of neurogenic inflammation (89). Neurogenic inflammation, driven by low-grade CGRP release, has been implicated in the inhibition of insulin secretion and T2D progression (5).

#### Air pollution, TRPV1, and autonomic imbalance

**Figure 3:**
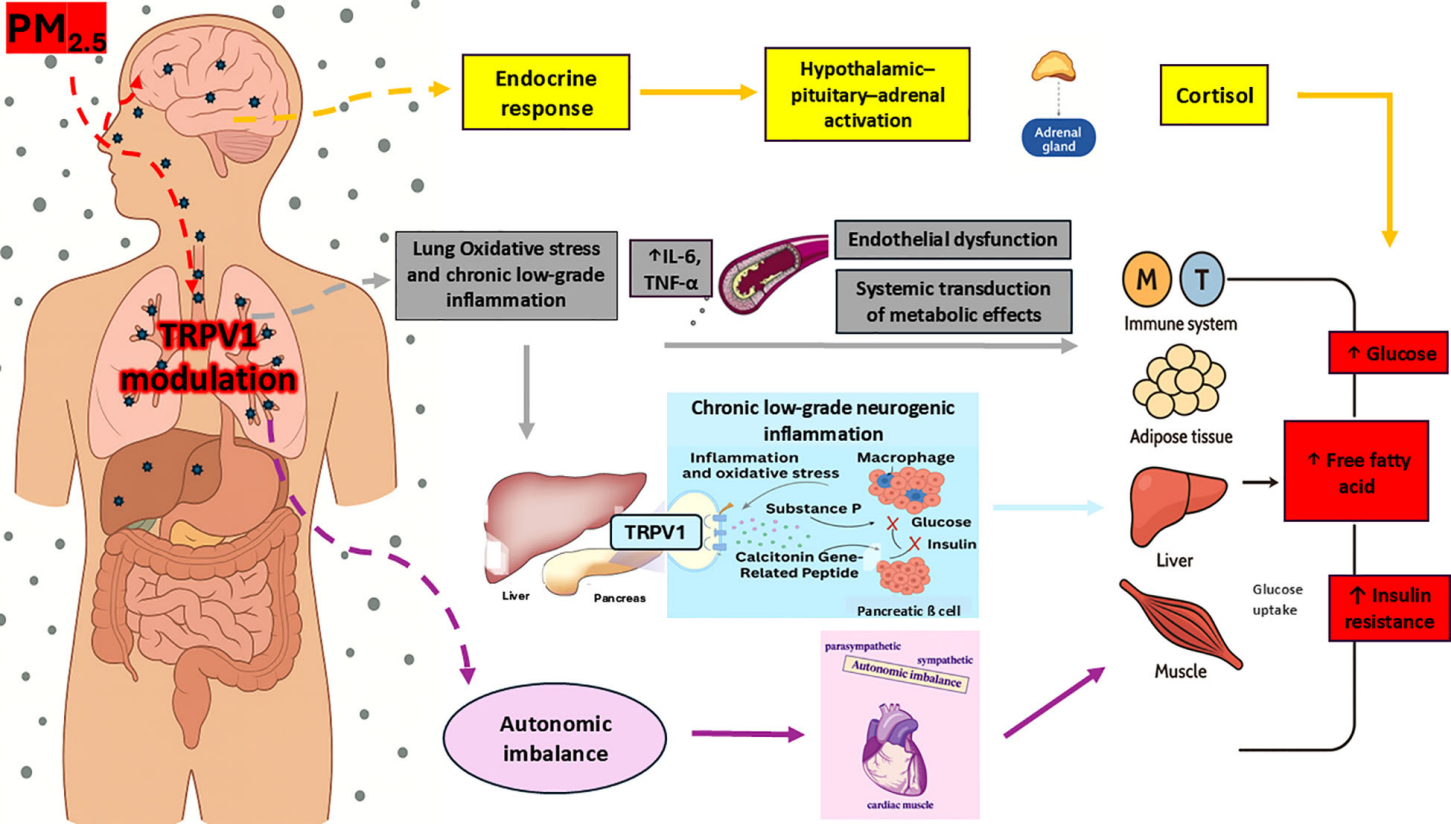
Mechanistic model of TRPV1-mediated pathways linking PM_2.5_ exposure to systemic metabolic disturbances. Adapted from Rajagopalan et al. (4) and Gram et al. (5). Upon inhalation, PM_2.5_ interacts with TRPV1 receptors located on bronchopulmonary vagal C-fiber endings. This modulation promotes lung low-grade inflammation and oxidative stress, leading to increased circulating proinflammatory cytokines levels like IL-6 and TNF-α. These promote endothelial dysfunction and contribute to the systemic propagation of inflammatory signals. The resulting inflammation affects multiple peripheral target organs—including the immune system, adipose tissue, skeletal muscle, liver, and pancreas. Within the pancreas, inflammatory mediators may modulate TRPV1-expressing sensory neurons, leading to local neurogenic inflammation. This is mediated by the release of CGRP and SP, which contribute to β-cell dysfunction, impaired insulin secretion, and insulin resistance. Additionally, pulmonary TRPV1 modulation may contribute to autonomic imbalance, characterized by increased sympathetic nervous system activity. This shift in autonomic tone impairs glucose and energy regulation and further amplifies inflammation-driven metabolic dysfunction. Finally, PM_2.5_ may also reach the central nervous system directly via the olfactory nerve. This route can trigger hypothalamic inflammation and activate the HPA axis, resulting in elevated cortisol release and further endocrine-metabolic disturbances. TRPV1, Transient receptor potential vanilloid 1; CGRP, Calcitonin gene-related peptide; SP, Substance P; HPA, Hypothalamic–pituitary–adrenal axis.

Air pollution, particularly PM_2.5_ exposure, activates the SNS, increasing stress hormone production, blood pressure, and impairing glucose metabolism (4). DEPs a specific type of particulate matter emitted from diesel engines and an important contributor to urban PM_2.5_— (75), directly stimulate TRPV1 activity (90), exacerbating SNS overactivity and neurogenic inflammation. This autonomic imbalance, combined with oxidative stress and inflammation, creates a proinflammatory environment that promotes insulin resistance and increases T2D risk (4, 73). **Figure 3** illustrates how TRPV1 acts as a key mediator in the translation of PM_2.5_ exposure into neuroimmune and metabolic dysfunction, integrating pulmonary, autonomic, and endocrine pathways.

#### Polymorphisms and sensitivity

TRPV1 is a highly polymorphic channel, with genetic variants influencing its sensitivity to stimuli such as capsaicin and inflammatory agents (91). These polymorphisms may affect pain perception, metabolic regulation, and susceptibility to conditions such as diabetes and cardiovascular diseases, particularly in individuals chronically exposed to PM_2.5_. Notably, DEPs directly activate TRPV1: DEPs exposure enhances capsaicin-evoked Ca²^+^ influx in TRPV1-expressing cells and TRPV1 antagonism abolishes DEP-induced cardiovascular effects *in vivo* (90, 92). Heterologous expression studies further show TRPV1 activation by particulate matter, with responses ranked Coal Fly Ash (CFA1) ≥ diesel exhaust PM ≥ silica, indicating DEPs behaves as an agonist (93). Consequently, carriers of TRPV1 variants that increase channel sensitivity may experience amplified biological effects of DEP-rich PM_2.5_, potentially elevating their risk for metabolic and cardiovascular disorders.

#### TRPV1 in autonomic dysfunction

In addition to the multi-level evidence of a direct interaction between DEPs and TRPV1 (90, 92, 93), human studies have linked TRPV1 to autonomic dysfunction in patients with diabetes and metabolic syndrome (MetS). For instance, adolescents with diabetic autonomic neuropathy (DAN) exhibit reduced capsaicin cough reflex sensitivity (CRS) and decreased HRV, suggesting the presence of early autonomic impairment (94). Evidence from MetS patients shows increased capsaicin CRS associated with elevated FBG, further supporting a role for glucose dysregulation in autonomic dysfunction (95). In addition, early T2D rat models, hyperglycemia-induced protein kinase C (PKC) activation sensitized TRPV1 in skeletal muscle afferents, amplifying exercise-induced blood pressure responses (96). These findings suggest that TRPV1 contributes to cardiovascular risk in T2D patients and could be a promising therapeutic target.

#### Therapeutic potential of TRPV1 antagonists

TRPV1 antagonists offer a novel therapeutic approach for managing T2D. By inhibiting CGRP-containing neuron overactivation, these drugs may reduce neurogenic inflammation, increase insulin secretion, and improve glucose metabolism. Preclinical studies demonstrated that genetic inactivation of TRPV1 in male C57BL/6J mice improved glucose tolerance and insulin secretion, while pharmacological blockade in Zucker obese rats reduced inflammatory markers in adipose tissue and ameliorated glucose metabolism (66). Additionally, TRPV1 antagonism has been shown to decrease hepatic gluconeogenesis and enhance energy expenditure by promoting the browning of white adipose tissue, mechanisms that further support its metabolic benefits (97). Currently, XEN-D0501, a TRPV1 antagonist, is undergoing clinical trials to assess its efficacy in regulating blood glucose and mitigating T2D-related inflammatory complications (98). TRPV1 serves as a critical mediator linking PM_2.5_ exposure to T2D through mechanisms involving neurogenic inflammation, autonomic dysfunction, and metabolic disruption. Its therapeutic potential makes TRPV1 a promising target for innovative treatments addressing both metabolic and inflammatory aspects of T2D. Further research is essential to fully elucidate the role of TRPV1 and optimize interventions for individuals chronically exposed to air pollution.

## Conclusions

The association between T2D and PM_2.5_ exposure is supported by an emerging body of evidence, albeit with variability in strength and consistency across studies. While the associations may be characterized as weak in some studies, the hypothesis that PM_2.5_ pollution, pervasive in urban environments, acts as an additional risk factor for T2D remains plausible. Given the vast number of individuals exposed to PM_2.5_, even marginal effects could translate into a significant public health burden. Multiple mechanisms are involved in the development or worsening of T2D due to PM_2.5_ exposure, including low-grade systemic inflammation, oxidative stress, endothelial dysfunction, chronic neurogenic and hypothalamic inflammation, as well as autonomic imbalance. Among these, autonomic imbalance seems to play a particularly important role, in increasing the vulnerability of individuals with T2D to the acute cardiovascular effects of PM_2.5_, through enhanced sympathetic activity and reduced HRV. This hypersusceptibility aligns with the inflammatory nature of T2D, which is characterized by persistent low-grade inflammation and excessive ROS production. These factors potentially enhance the proinflammatory and oxidative stress responses triggered by PM_2.5_ exposure. Moreover, ongoing neurogenic inflammation may contribute to altered autonomic regulation, further increasing the risk of cardiac complications. Recent insights have also revealed the involvement of TRPV1, a non-selective cation channel directly sensitized by key components of air pollution, as a probable mediator of neurogenic inflammation and metabolic disruptions, providing a novel target for intervention.

### Current limitations and uncertainties

Numerous uncertainties persist owing to methodological variability, population heterogeneity, and divergent study designs, which complicate the interpretation of findings across the epidemiological literature. While most studies demonstrate a positive association between PM_2.5_ exposure and T2D, the strength and consistency of these associations vary considerably. A key source of heterogeneity lies in how PM_2.5_ exposure is assessed. Studies rely on a variety of methods, such as individual-level monitoring, fixed-site stations, satellite-based modeling, and land-use regression, which differ substantially in spatial and temporal resolution. These differences can lead to exposure misclassification, particularly when area-level averages are used in place of fine-scale, individual-level estimates. In urban environments characterized by complex pollution gradients, such misclassification can dilute associations and bias risk estimates toward the null. Another major limitation concerns coexposure to other pollutants. Many studies do not adequately adjust for additional ambient contaminants, such as nitrogen dioxide (NO_2_) or ozone, which may also contribute to metabolic dysfunction. The lack of multipollutant models makes it difficult to isolate the specific role of PM_2.5_, and raises the possibility of residual confounding (99). Furthermore, inconsistency in the control of confounding variables, especially behavioral and metabolic risk factors such as body mass index, diet, physical activity, socioeconomic status, and comorbidities, may lead to biased or imprecise estimates. These variables are not only risk factors for diabetes but also influence exposure patterns, for example through residential location. In several studies, effect modification by sex, age, or obesity status has been reported, highlighting the importance of stratified analyses to detect differential susceptibility. Variability in outcome ascertainment also contributes to heterogeneity. While some studies use self-reported physician diagnoses, others rely on clinical markers (e.g., fasting glucose, HbA1c) or administrative data. These differences can affect both the sensitivity and specificity of case identification, particularly in detecting undiagnosed or prediabetic individuals. Additionally, in time-series and panel designs, heterogeneity may arise from inconsistent choices of lag periods and the limited assessment of harvesting effects. Such temporal misalignments can complicate the causal interpretation of short-term associations between PM_2.5_ exposure and glycemic outcomes (100). Finally, differences in study design, including cross-sectional versus longitudinal approaches, sample size, and duration of follow-up, may influence the robustness and comparability of results. Although publication bias cannot be excluded, particularly in smaller studies with null results, recent meta-analyses consistently report positive associations, reinforcing the plausibility of a causal link. Standardized methodologies, harmonized confounder adjustment strategies, and improved exposure modeling are therefore essential to reduce heterogeneity and strengthen the evidence base. Animal experimental evidence also carries important limitations. Most rodent studies use acute intratracheal boluses of supra-ambient DEP doses, are restricted to a single species/sex, and are conducted in isolated or anesthetized preparations; TRPV1 involvement is typically inferred from a single antagonist without genetic confirmation. These features together with species-specific differences in particle deposition, TRPV1 expression/regulation, and autonomic circuitry limit direct translation to humans chronically exposed to PM_2.5_.

### Policy implications

Addressing the health impacts of PM_2.5_ requires comprehensive strategies, including effective monitoring, through enhanced air quality monitoring to identify high-risk areas and periods, enabling proactive interventions, reduction policies, such as stricter emission regulations, promotion of clean energy, and urban planning to minimize PM_2.5_ exposure, and targeted interventions aimed at educating vulnerable populations, such as children, elderly individuals, and individuals with T2D, on protective measures. In addition, research initiatives involving controlled studies are needed to better understand dose–response relationships and the specific effects of PM_2.5_ on diabetes progression and severity.

### Future research directions

To fully elucidate the complex relationship between PM_2.5_ exposure and T2D, future research must address several key gaps. First, the direct effects of PM_2.5_ on pancreatic β-cell integrity, counterregulatory hormones such as glucagon, and insulinotropic signaling pathways remain poorly understood. Second, central mechanisms, including hypothalamic circuits regulating appetite and satiety, as well as autonomic nervous system dysfunction, may mediate systemic metabolic and inflammatory responses and warrant further investigation. Third, studies should better characterize differential susceptibility in vulnerable populations, particularly individuals at various stages of diabetes progression. Specifically, future efforts should prioritize the functional role of TRPV1 in human populations, a deeper molecular and toxicological characterization of PM_2.5_ chemical constituents, and the establishment of precise dose–response relationships stratified by age, sex, and metabolic phenotype.

Human-derived organoids (pancreatic islets, hypothalamic and autonomic neuronal organoids) exposed to chemically characterized PM_2.5_/DEPs fractions could have the potential to clarify TRPV1-dependent, cell-specific mechanisms and bridge animal and clinical findings. Using organoids with different TRPV1 genotypes, or modulating TRPV1 pharmacologically, allows testing gene–environment interactions in a controlled human system. These platforms complement *in vivo* models by adding human relevance while retaining experimental manipulability.

PM_2.5_ exposure represents a modifiable risk factor contributing to the morbidity and mortality associated with T2D and its cardiovascular complications. While the precise mechanisms remain incompletely understood, the available evidence underscores the urgency of implementing air pollution reduction policies and advancing targeted research to mitigate the global health burden of T2D.
